# Increased threshold of short-latency motor evoked potentials in transgenic mice expressing Channelrhodopsin-2

**DOI:** 10.1371/journal.pone.0178803

**Published:** 2017-05-31

**Authors:** Wei Wu, Wenhui Xiong, Ping Zhang, Lifang Chen, Jianqiao Fang, Christopher Shields, Xiao-Ming Xu, Xiaoming Jin

**Affiliations:** 1 Department of Neurological Surgery, Stark Neuroscience Research Institute, Indiana University School of Medicine, Indianapolis, Indiana, United States of America; 2 Spinal Cord and Brain Injury Research Group, Stark Neuroscience Research Institute, Indiana University School of Medicine, Indianapolis, Indiana, United States of America; 3 Department of Anatomy and Cell Biology, Stark Neuroscience Research Institute, Indiana University School of Medicine, Indianapolis, Indiana, United States of America; 4 Norton Neuroscience Institute, Norton Healthcare, Louisville, Kentucky, United States of America; 5 Department of Acupuncture, Third Affiliated Hospital of Zhejiang Chinese Medical University, Hangzhou, Zhejiang Province, China; 6 Zhejiang Chinese Medical University, Hangzhou, China; Nanjing Normal University, CHINA

## Abstract

Transgenic mice that express channelrhodopsin-2 or its variants provide a powerful tool for optogenetic study of the nervous system. Previous studies have established that introducing such exogenous genes usually does not alter anatomical, electrophysiological, and behavioral properties of neurons in these mice. However, in a line of Thy1-ChR2-YFP transgenic mice (line 9, Jackson lab), we found that short-latency motor evoked potentials (MEPs) induced by transcranial magnetic stimulation had a longer latency and much lower amplitude than that of wild type mice. MEPs evoked by transcranial electrical stimulation also had a much higher threshold in ChR2 mice, although similar amplitudes could be evoked in both wild and ChR2 mice at maximal stimulation. In contrast, long-latency MEPs evoked by electrically stimulating the motor cortex were similar in amplitude and latency between wild type and ChR2 mice. Whole-cell patch clamp recordings from layer V pyramidal neurons of the motor cortex in ChR2 mice revealed no significant differences in intrinsic membrane properties and action potential firing in response to current injection. These data suggest that corticospinal tract is not accountable for the observed abnormality. Motor behavioral assessments including BMS score, rotarod, and grid-walking test showed no significant differences between the two groups. Because short-latency MEPs are known to involve brainstem reticulospinal tract, while long-latency MEPs mainly involve primary motor cortex and dorsal corticospinal tract, we conclude that this line of ChR2 transgenic mice has normal function of motor cortex and dorsal corticospinal tract, but reduced excitability and responsiveness of reticulospinal tracts. This abnormality needs to be taken into account when using these mice for related optogenetic study.

## Introduction

Neurons that are genetically modified to express light-sensitive channels allow precise optogenetic activation or inhibition of their activities, providing a powerful tool for studying neural network under physiological and pathological conditions. Transgenic mice that express Channelrhodopsin-2 (ChR2) or its variants under the control of a specific promoter have been widely used for *in vitro* and *in vivo* studies of the nervous system at cellular, circuit, and behavioral levels [1–3]. Particularly, Thy1-ChR2 transgenic mice (Thy1-ChR2-EYFP lines 9 and 18) are the first lines that express transgenes in subsets of neurons throughout the nervous system [1, 2].

Previous studies showed that transgenic mice often maintain unaltered structural and functional properties. For example, there are no significant differences in morphological features and electrophysiological properties between GFP-positive and GFP-negative hippocampal dentate granule cells of Thy1-GFP transgenic mice [4]. ChR2 expressing cortical neurons of Thy1-ChR2 mice show no significant changes in intrinsic properties, including resting membrane potential, input resistance, and properties of action potential [2]. Similarly, hippocampal neurons with viral expression of ChR2 for at least one week maintain normal intrinsic electrophysiological properties [5]. Specific expression of ChR2 at axon initial segments by using ankyrinG-binding loop of voltage-gated sodium channels does not cause significant changes in neuronal passive and active electrical properties such as action potential threshold and spike number induced by current injection in these neurons [6]. These observations may lead to an assumption that transgenic mice usually maintain their “normal” structural and functional properties. For neurons that express ChR2 or its variants, most attention has been focused on characterizing electrophysiological properties of the introduced photosensitive currents. However, it has been shown that long-term high expressions of ChR2-YFP through *in utero* electroporation or viral infection causes abnormal neuronal morphologies including swelling axons, formation of cylinders that envelope pyramidal neuron dendrites and axons, and spherical structures surrounding neuronal somata [7]. Although such structural alterations occur only in neurons that strongly express ChR2 for a long time, whether such morphological abnormalities are accompanied or preceded by functional alterations in ChR2-expressing neurons is unknown.

In our recent study using the line 9 Thy1-ChR2 transgenic mice, we accidentally found that short-latency (~5 ms) motor evoked potentials (MEPs) induced by transcranial magnetic stimulation (TMS) and transcranial electrical stimulation had dramatically reduced amplitude and higher threshold. In contrast, long-latency MEP evoked by electrical stimulation of the motor cortex remained intact. Patch clamp recording and motor behavioral assessments indicate no significant changes in cellular electrophysiological properties of cortical pyramidal neurons and motor behavior. The results suggest that ChR2 expression in this transgenic line has a specific effect on the motor pathway involving reticulospinal tract that generates short-latency MEPs.

## Materials and methods

### Animals

Transgenic mice expressing ChR2-YFP under the control of Thy1 promoter (line 9, stock number 7615, Jackson lab) and C57BL wild type (WT) sibling mice of the same background were used in the experiments. The mice were between 8–10 weeks old. All procedures were approved by the Animal Care and Use Committee of the Institutional Guide for the Care and Use of Laboratory Animals at Indiana University School of Medicine.

### MEPs induced by TMS and percutaneous electrical stimulation

Eight C57BL mice and 13 Thy1-ChR2 transgenic mice were used for recording TMS-evoked MEPs. TMS-induced MEPs were obtained by stimulating awake, non-anesthetized, restrained mice as described previously [8, 9]. Briefly, mice were restrained in thin and porous stockinet on a 4.5″ x 2″ wooden board with thumb tacks. Hindlimbs were exposed to enable insertion of recording electrodes into the gastrocnemius muscles bilaterally, with the active electrodes being placed in the muscle belly and the reference electrodes being placed near the distal tendon. A ground electrode was placed subcutaneously between the coil and recording electrodes. A 5 cm diameter, single round electromagnetic coil without casing was connected to a Cadwell MES-10 stimulator (Cadwell Laboratories, Kennewick, WA). The coil was placed on the skull of the animal tangentially, with the region of maximal output being aimed at the sensorimotor cortex. MEPs were elicited by activating cortical and subcortical structures with the coil being placed over the mouse’s scalp and positioned for maximal activation. A single magnetic pulse at 90% of maximal output intensity (2 Tesla peak output) was used. If a response could not be evoked in ChR2 transgenic mice, the maximal TMS intensity was applied and the stimulation was repeated three times. Depending on the amplitude of the responses, a gain of x5000 was used to record the compound muscle action potentials. Responses were recorded at intervals of 1 min. All animals were tested weekly for 5 weeks. In both WT and ChR2 groups, the mean amplitudes and latencies were calculated based on responses to 90% stimulation intensity.

For recording MEPs evoked by transcranial electrical stimulation, 8 C57BL mice and 9 Thy1-ChR2 mice were anesthetized with ketamine (100 mg/kg, i. p.). Previous studies showed that ketamine does not significantly affect the amplitude and waveform of MEPs in dogs and rodents [10, 11]. A 9 mm cup filled with Electro-conductive gel (Tag Gel, Pharmaceutical Innovations, Newark, NJ) was placed on the vertex of the animal scalp as an anode. The original clip part of the ear electrode was shortened and wired to serve as a cathode that contacted the hard palate of the animal behind the incisor. Electrical stimulation was applied to excite the brain using a stimulator (Digitimer DS7A, Digitimer, Garden City, UK). A single pulse of stimulation (100 μs, 350 V) was delivered via a modified E5-9S ear electrode (Electro-cap Inc. Eaton, OH).

All MEP data were analyzed offline using Clampfix software. Events that occurred within 4–8 ms after TMS and had peak amplitude two standard deviations of the baseline activity were regarded as MEPs. Latencies for each sweep were measured from the onset of the TMS artifact to the beginning of the evoked-events. The amplitudes of the evoked MEPs were measured from peak to peak. Since MEPs could not be evoked even with maximal TMS in the majority of ChR2 mice, mean MEP amplitudes and latencies in this group were calculated only from responses in which MEPs were successfully induced.

### MEPs induced by cortical electrical stimulation

MEPs were recorded using cortical electrical stimulation in 6 ChR2 transgenic mice and 6 WT mice. After the animals were anesthetized with ketamine (100 mg/kg, i.p.), stainless steel screws (2.5 mm long and 1.2 mm in diameter) were implanted onto the skull above the primary motor cortex. A needle electrode was inserted into contralateral gastrocnemius muscle for recording MEPs. Electric shocks were delivered with a duration of 200 μs and intensities between 0.4–1.5 mA.

### Behavior assessment

All behavior analyses were conducted according to previously published methods by two blinded observers who had no knowledge of group assignment.

*Basso Mouse Scale (BMS)*: This open-field locomotor scoring system consists of scores from 0 (no ankle movement) to 9 (frequent or consistent plantar stepping, mostly coordinated, paws parallel at initial contact and lift off, and normal trunk stability and tail always up) [12].

*Rotarod test*: This test was used to assess motor coordination, balance, and motor learning. The mice were placed on a single lane rotarod according to our existing protocol [13]. The speed was set to a constant acceleration from 0–18 round per minute (rpm) or from 0–30 rpm for a total of 120 seconds. The latency to fall from the rod was recorded. Each mouse was individually scored for 3 trials and the scores were averaged to generate a final score for each session.

*Grid-walking*: The mice were placed on a horizontal grid, and allowed to walk freely on the grid for 3 minutes [14]. The total steps and falls of each hindlimb were recorded.

### Slice preparation and patch clamp recording

Brain slices were prepared using previously described procedures [15, 16]. Following deep anesthetization with pentobarbital (55 mg/kg, i.p.), the mice were decapitated. Coronal cortical slices containing the motor cortex were cut with a vibratome (Leica VT1200, Leica) in ice cold (4°C) oxygenated slicing solution containing (in mM) 230 sucrose, 2.5 KCl, 1.25 NaH_2_PO_4_, 10 MgSO_4_.7HO_2_, 10 glucose, 0.5 CaCl_2_.2H_2_O, and 26 NaHCO_3_. The slices were incubated at 34°C for 1 hour in standard artificial cerebrospinal fluid (ACSF), and were then kept at room temperature. The ACSF contained (in mM) 126 NaCl, 2.5 KCl, 1.25 NaH_2_PO_4_, 2 CaCl_2_, 2 MgSO_4_.7H_2_O, 26 NaHCO_3_, and 10 glucose; pH 7.4 when saturated with 95% O_2_-5% CO_2_.

Patch clamp recordings were made in a heated chamber at 35°C from YFP-expressing layer V pyramidal neurons of ChR2 mice or layer V pyramidal neurons of WT mice. Neurons in slices were visualized under infrared DIC illumination, which did not noticeably induce light-activated current. Patch electrodes were pulled from borosilicate glass tubing (1.5 mm OD), and had an impedance of 3–5 MΩ when filled with intracellular voltage clamp solution containing (in mM): 20 KCl, 100 cesium gluconate, 10 HEPES, 4 Mg-ATP, 0.3 Na-GTP, 10 sodium phosphocreatine and 3 QX-314. For current clamp recordings, K-gluconate based solution was used, which contained (in mM): 100 K-gluconate, 20 KCl, 10 HEPES, 4 Mg-ATP, 0.3 NaGTP and 10 sodium phosphocreatine. To measure spontaneous action potential (AP) firing rates, current clamp recordings were made by adjusting the membrane potentials to -60 mV and in a modified ACSF that slightly enhanced neuronal excitability. The modified ACSF contained (in mM): 124 NaCl, 3.5 KCl, 0.5 MgCl_2_, l.25 NaH_2_PO_4_, 26 NaHCO_3_, 1 CaCl_2_, and 25 Dextrose [17].

Neurons with a resting membrane potential below -60 mV and a series resistance of less than 15 MΩ were included in data analysis. Intrinsic properties and AP firings were determined from neuronal responses to a series of 500 ms hyperpolarizing and depolarizing currents pulses (25–50 pA steps) under current clamp mode. Spike frequencies in response to step current injections (I-F curve) were constructed. Analysis of intrinsic properties was done using Clampfit 9.0 software. Resting membrane potential was measured as the membrane voltage after break-in and no current was injected. Input resistance was determined from the slope of a best-fit-line through the linear segment of a voltage-current relationship. The first AP elicited by lowest current injection was used for measuring AP properties. AP threshold was determined at the voltage level when voltage deflection exceeded 10 mV/ms. AP duration was measured at its half maximal amplitude, while AP amplitude was measured as the difference between AP threshold and the peak amplitude.

### Statistical analysis

Data are presented as mean ± SEM. Statistical analyses were performed using Microsoft Excel and OriginPro 8 software. Statistical significance was determined using Student *t*-test and one-way ANOVA followed by Tukey’s test, with a *p* value of *p* < 0.05.

## Results

### Reduced short-latency MEPs induced by TMS and percutaneous electrical stimulation in ChR2 mice

**Fig 1:**
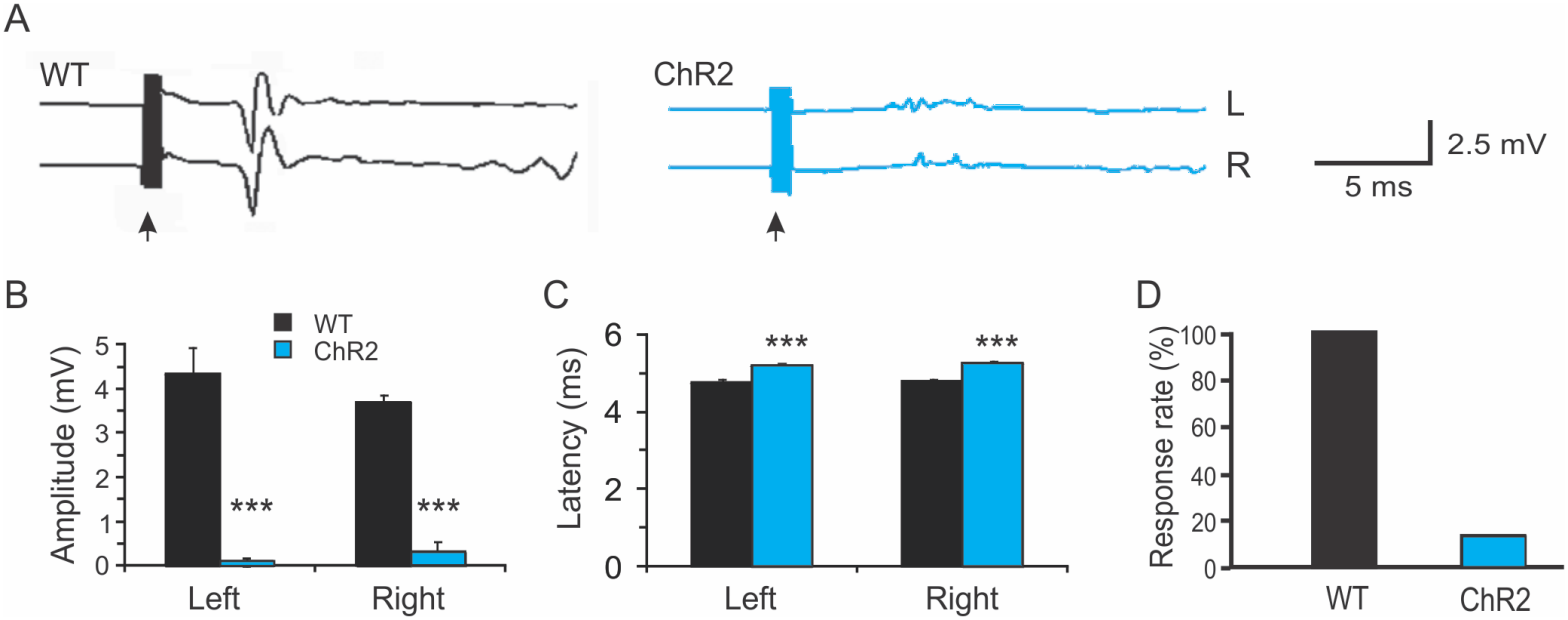
Reduced motor evoked potentials induced by transcranial magnetic stimulation in Thy1-ChR2 transgenic mice. A. Representative traces showing bilateral TMS-evoked motor evoked potentials (MEPs) in a wild type C57 Black mouse (WT; black) and a Thy1-ChR2 transgenic mouse (blue). The black arrows indicate times of transcranial magnetic stimulation (TMS). B-D. The amplitudes of bilateral TMS-evoked MEPs were dramatically decreased in the ChR2 mice compared with WT mice (B); the latencies of bilateral TMS-evoked MEPs were significantly increased in the ChR2 mice (C), and the percentage of mice in which MEPs were induced by TMS was greatly reduced in the ChR2 mice (D) (***: *p*<0.001, Student *t*-test).

TMS reliably induced bilateral EMG responses in all WT animals (Fig 1A and 1D, 100%, n = 8). The mean onset latency of the TMS-induced MEPs was 4.82 ± 0.14 ms, and the amplitude was 4.07 ± 1.02 mV (Fig 1A–1C). In contrast, MEPs were induced in only a small percentage of ChR2 transgenic mice (Fig 1D. 15%, n = 13). In the ChR2 mice that failed to respond to TMS, neither increasing the TMS output from 90% to the maximum nor changing coil position and orientation was able to induce any response. We calculated onset latency and amplitude in ChR2 mice in which discernable MEPs were recorded. Their onset latency was significantly delayed compared with that of the WT mice (Fig 1A–1C, 5.12 ± 0.04 ms, *p*<0.001, Student *t*-test), and the amplitude was dramatically reduced (0.67 ± 0.23 mV, *p*<0.001). Thus, TMS induced smaller MEPs with longer onset latencies in ChR2 mice.

**Fig 2:**
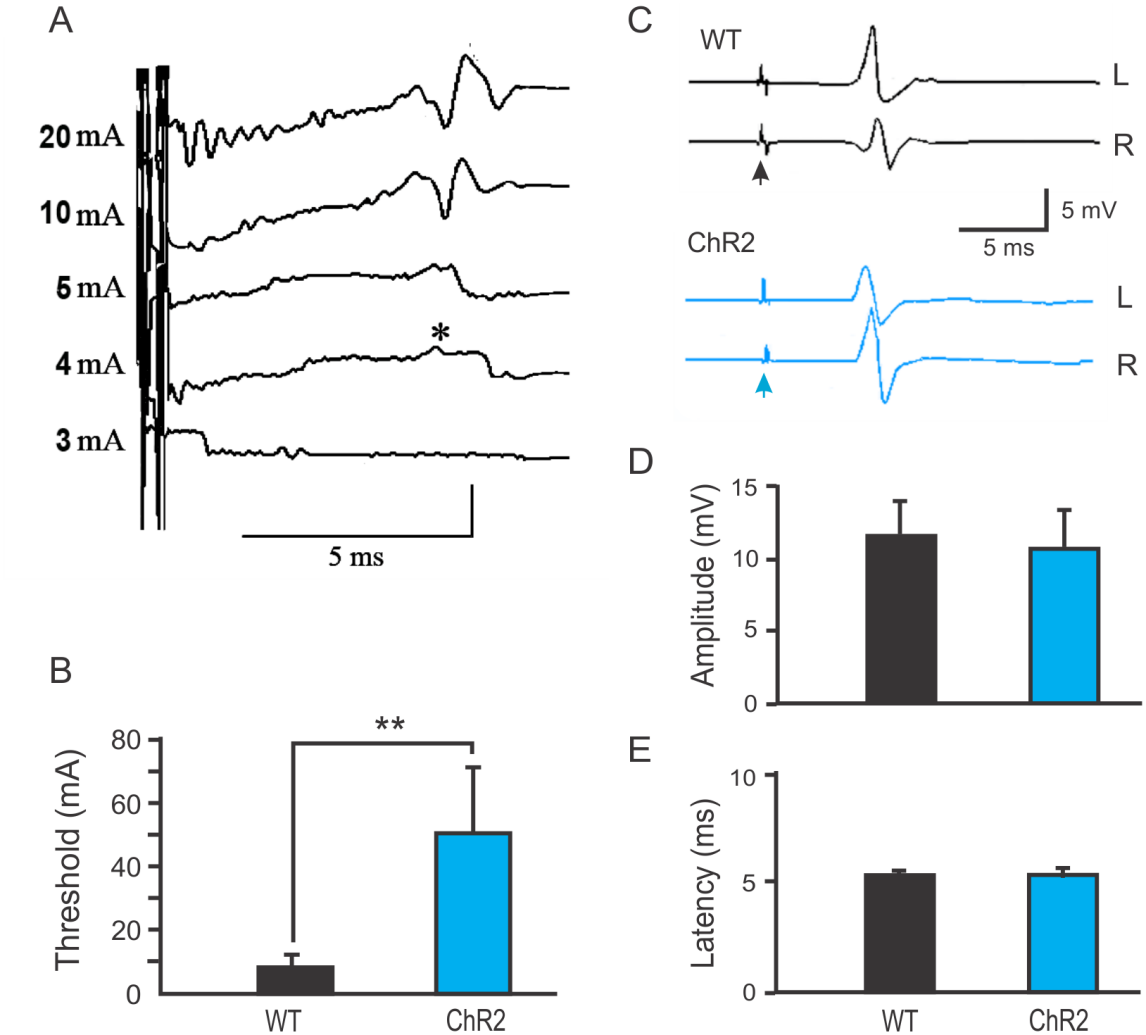
Reduced MEPs evoked by transcranial electrical stimulation in Thy1-ChR2 transgenic mice. A. Representative traces showing electrically evoked motor evoked potentials (MEPs) in a wild type C57 Black mouse (WT). The asterisk indicates a response evoked with an electrical pulse at the threshold level (4 mA). B. The threshold for electrically evoking MEPs was significantly higher in Thy1-ChR2 mice than in WT mice (**: *p* < 0.01, n = 8–9 mice in each group). C. Representative traces showing similar latency, amplitudes, and waveform of maximally evoked MEPs in WT (black) and ChR2 (blue) mice. The arrows indicate times of electrical stimulation. D-E. The amplitudes (D) and latency (E) of MEPs evoked at maximal electrical stimulation were similar between the WT and Thy1-ChR2 mice (*p* > 0.05).

To confirm the observed change in TMS-induced MEPs and differentiate between potential cortical and subcortical changes in the reduced MEPs, we recorded MEPs induced by transcranial electrical stimulation of the motor cortex (Fig 2A). The thresholds for evoking MEPs were 7.8 ± 4.9 and 46.2 ± 21.8 mA for the WT and ChR2 mice, respectively (Fig 2B, n = 8–9 in each group, *p*<0.01). However, there were no differences between the ChR2 and WT mice in the amplitude and latency of MEPs induced by maximal stimulus (Fig 2C–1E. 5.2 ± 0.14 ms vs. 5.2 ± 0.2 ms in latency, and 11.8 ± 2.5 mV vs. 10.9 ± 2.6 mV in amplitude for ChR2 and WT mice, respectively). The results suggest that ChR2 transgenic mice have a higher threshold for evoking MEPs through transcranial magnetic or electrical stimulation.

### No change in MEPs induced by cortical electrical stimulation in ChR2 mice

**Fig 3:**
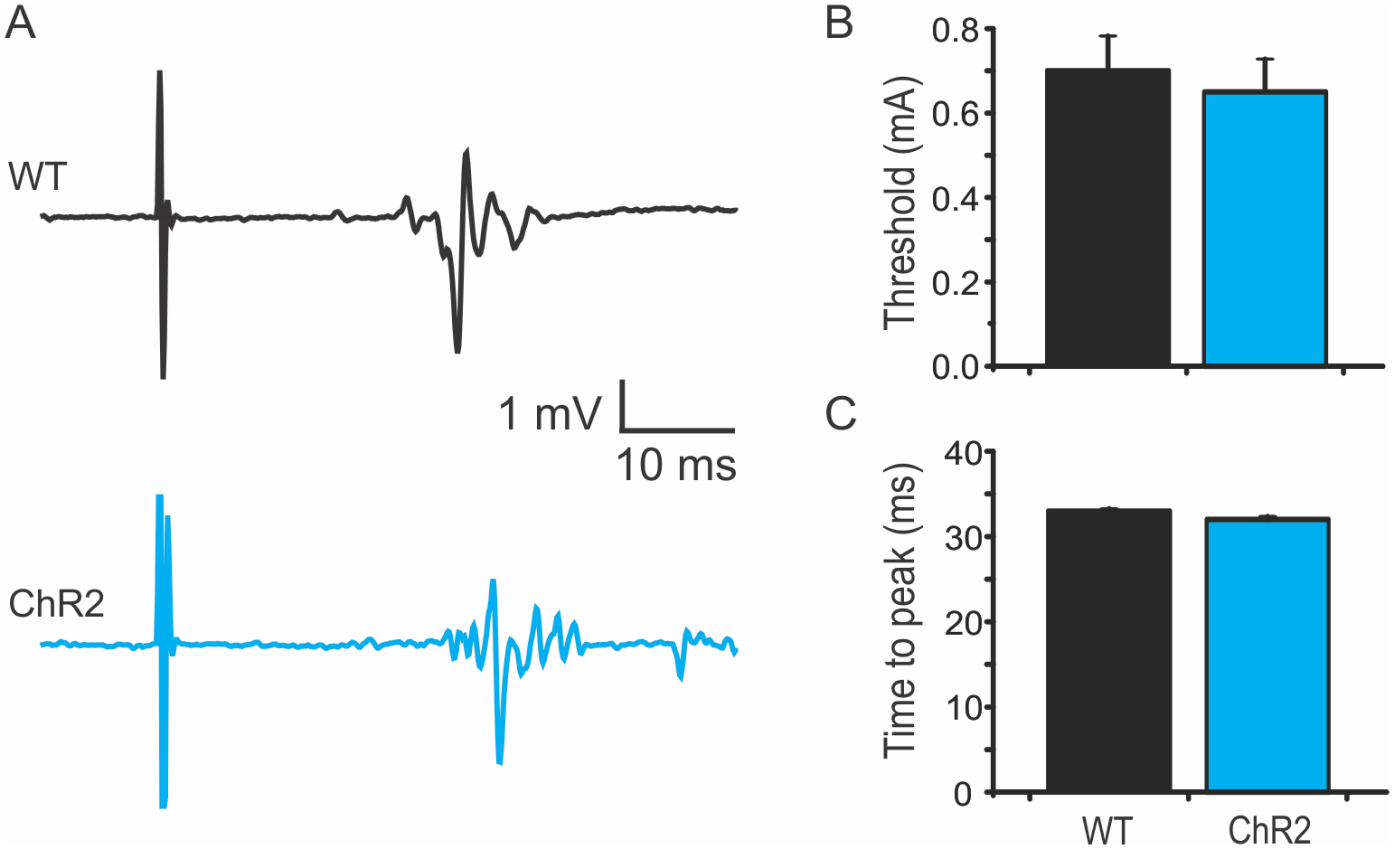
No significant change in MEPs induced by electrical stimulation of motor cortex in ChR2 transgenic mice. A. Representative traces showing similar long-latency motor evoked potentials (MEPs) in wild type (WT; black) and ChR2 (blue) mice. B-C. There were no significant differences in stimulating thresholds (B) and time to peak amplitude of MEP (C) between WT and ChR2 mice.

It has been shown that two types of MEPs can be evoked in rodents. The first is a short-latency and high amplitude (~ 5 ms) response; the second is a long-latency (~15 ms), polyphasic wave that involves motor cortex and corticospinal tract [18, 19]. To specifically evaluate the long-latency MEPs, mouse motor cortex was electrically stimulated via an implanted screw. The mean thresholds of the ChR2 mice and WT mice were 0.65 ± 0.08 mA and 0.70 ± 0.08 mA, and the times to MEP peak were 32.0 ± 0.36 ms and 33 ± 0.26 ms, respectively (Fig 3A–3C. n = 6 in each group). Thus, there are no differences in the threshold and latency of the long-latency MEPs between WT and ChR2 mice.

### Unaltered electrophysiological properties of cortical layer V pyramidal neurons in ChR2 mice

To determine the electrophysiological mechanism of the reduced MEPs evoked by transcranial magnetic and electrical stimulation in the Thy1-ChR2 mice, we made whole-cell patch clamp recordings from layer V pyramidal neurons of the motor cortex in these mice. These neurons express ChR2 protein and are involved in activating corticospinal tract and generating MEPs. We first recorded spontaneous firing of action potentials (APs) of these neurons in a modified ACSF solution that slightly enhances neuronal excitability and reveals their spontaneous activity *in vitro*, and found that the AP firing rate in the ChR2 mice at -60 mV was similar to that of the WT mice (data not shown).

**Fig 4:**
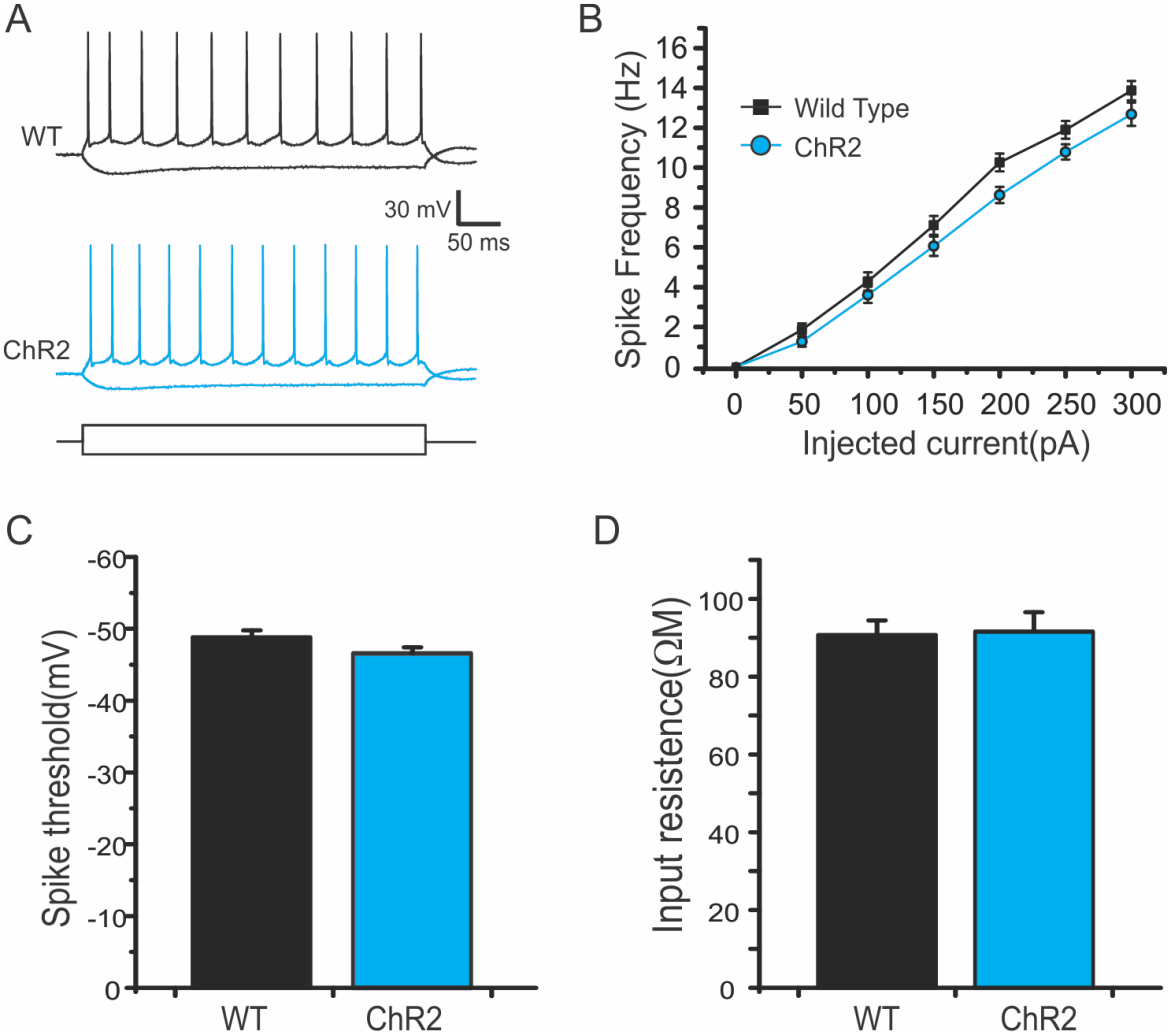
Similar intrinsic properties of layer V pyramidal neurons in WT and Thy1-ChR2 mice. A. Representative traces of action potential (AP) firing of layer V pyramidal neurons of wild type (WT; black) and ChR2 (blue) mice in response to current injections. B. Similar *I-F* slope in ChR2 mice: Spike frequencies to step current injections in ChR2 mice were similar to those of the WT mice (*p >* 0.05, one-way ANOVA). C-D. AP thresholds (C) and input resistances were similar between WT and ChR2 mice.

Under current clamp mode, we further recorded traces in response to injections of increasing current steps and plotted the relationship between the injected currents and frequencies of evoked spikes (I-F curve) (Fig 4A and 4B). The I-F curve from layer V pyramidal neurons of ChR2 mice had a similar slope as that of the WT mice (Fig 4B), suggesting that current injections induced similar frequency of AP spikes. There were also no significant differences in intrinsic membrane properties including resting membrane potential, input resistance, membrane time constant, action potential threshold, and AP half width and peak amplitude between the two groups (Table 1 and Fig 4C and 4D).

**Table 1:** Electrophysiological properties of membrane and action potential.

Group	n	V_m_ (mV)	R_Input_ (MΩ)	membrane time constant	AP threshold (mV)	AP amplitude (mV)	AP half width (ms)
**Wild type**	39	-64.7±2.1	90.7±3.8	23.6±1.4	-48.8±1.0	92.3±1.1	1.6±0.03
**Thy1-ChR2**	28	-63.3±0.8	91.6±6.0	25.1±1.2	-46.6±0.9	93.5±1.2	1.3±0.02

Abbreviations: AP, action potential; ChR2, channelrhodopsin-2

### No motor deficits in ChR2 mice

**Fig 5:**
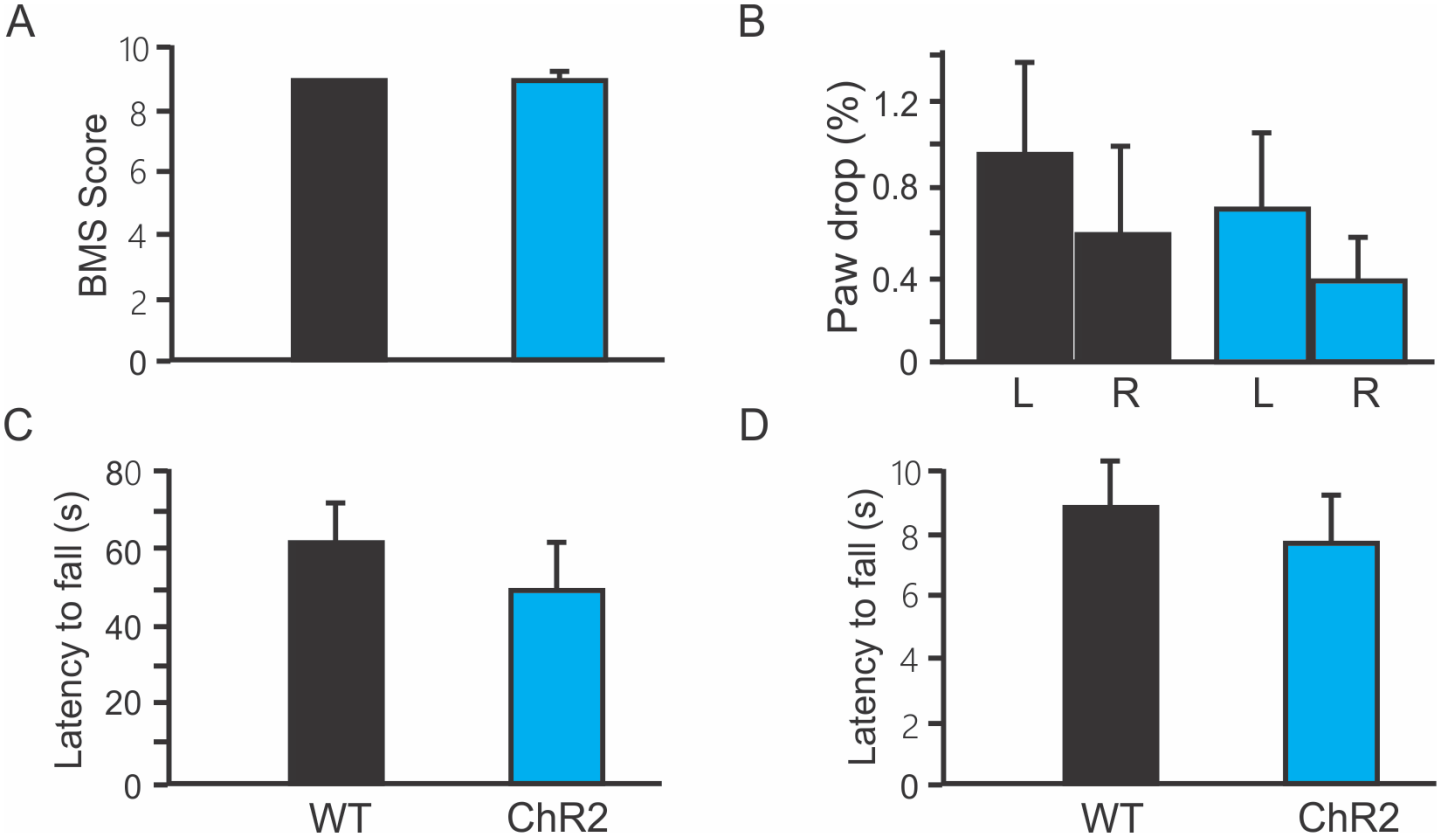
No significant change in motor function in Thy1-ChR2 transgenic mice. A. Basso Mouse Scale (BMS) locomotor scores were not different between wild type (WT) and ChR2 transgenic mice. B. Grid-walking test showed no significant differences in paw drop ratios of both left and right hindlimbs between WT and ChR2 mice. C-D. Rotarod test showed that there were no significant differences in the latency to fall at the speeds of 18 rpm (C) and 30 rpm (D) between WT and ChR2 mice.

To further explore whether a reduction in short-latency MEPs would cause any motor functional deficits, we assessed motor behavior in WT and ChR2 transgenic mice. There were no significant differences in BMS scores (Fig 5A. WT: 9; ChR2: 8.9 ± 0.3), ratios of paw drop in grid-walking test (Fig 5B. WT: 0.94 ± 0.4, ChR2: 0.7 ± 0.3 for left hindlimb; WT: 0.58 ± 0.4, ChR2: 0.4 ± 0.2 for right hindlimb), and latencies to fall in rotarod test (Fig 5C and 5D. WT: 88 ± 15 s; ChR2: 76 ± 15 s at the speed of 18 rpm; WT: 61 ± 10 s; ChR2: 50 ± 10 at the speed of 30 rpm). These results indicate normal motor function in ChR2 mice.

## Discussion

In the current study, we found a great decrease of amplitude and an increase of threshold in short-latency MEPs evoked by transcranial magnetic and electrical stimulations, but no change in long-latency MEPs evoked by cortical electrical stimulation in line 9 Thy1-ChR2 transgenic mice. Whole cell recordings indicated no changes in the intrinsic properties and excitability of layer V pyramidal neurons of the motor cortex in these mice. Motor behavioral assessments also indicate no deficits in motor function. The results indicate specific impairment of short-latency MEPs that are closely related to the function of reticulospinal tracts [20].

TMS is a well-established non-invasive brain stimulation technique that is useful for studying brain excitability and plasticity in human and animals [21–23]. Our initial findings of reduced TMS-induced MEPs in ChR2 mice are likely due to a lower efficacy of the TMS than that of direct cortical electrical stimulation. TMS induces MEPs via a magnetic coil and usually has low spatial precision. This may be particularly true when it is used for activating small mouse brains [24, 25]. However, it was this low efficacy of TMS that helped reveal the reduced motor response in ChR2 mice *in vivo*. This was confirmed by the increase in the threshold of MEP evoked by transcranial electrical stimulation. Additionally, the similar MEP amplitudes and latencies in response to strong percutaneous electric stimulation in WT and ChR2-expressing mice (Fig 2C–2E) suggest that reduced brain excitability, rather than possible abnormalities in descending motor tracts or neuromuscular coupling, is responsible for the reduced TMS-induced MEPs.

Previous studies have characterized two types of MEPs that are different in waveform, latency, and the involved motor pathways. The short-latency (5–7.5 ms) MEP has high amplitude and brainstem origin. It likely involves reticulospinal tracts, because transection of the midlateral funiculus severely compromises this component but transection of the rubrospinal tract in dorsolateral funiculus does not [19, 26, 27] [20]. In contrast, the long-latency MEPs appear as a set of polyphasic waves of variable latency (15–22 ms), with a prominent peak at latency around 15 ms and mainly involves corticospinal tracts. Transecting the motor cortex or dorsal corticospinal tract abolishes most of the long-latency MEPs but spares the short-latency MEPs [18–20]. Our result of reduced amplitude of short-latency MEPs indicates reduced excitability of reticulospinal tract in ChR2-YFP transgenic mice. Earlier studies also showed no clear correlation between the amplitude of short-latency MEPs and functional recovery in rats after spinal cord injury [28], which may explain the observation of no significant changes in motor behavior in ChR2 mice that have reduced MEP response.

In contrast to corticospinal and rubrospinal tracts which control fine motor movement, the reticulospinal tract plays an major role in eliciting locomotion that coordinates rhythmic stepping movement ([29–31]. It originates from dispersed nuclei in the reticular formation of the brainstem. An increased threshold of short-latency MEPs without changes in latency, waveform, and amplitude at maximal electrical stimulation (Fig 2B–2E) likely suggests reduced excitability of reticular nuclei instead of impaired axonal tracts. At the cellular level, an increase in AP threshold of these neurons may contribute to the observed increase in MEP threshold. Because a high density of voltage-gated sodium channels in axon initial segment (AIS) is essential for AP initiation [32], one explanation is that expression of exogenous ChR2 channels may reduce the expression or localization of endogenous voltage-gated sodium channels at the AIS. While it is unclear why reticulospinal neurons are specifically or more prominently affected by ChR2 expression, several factors may potentially contribute to such specific effect. These neurons are a group of brainstem locomotion neurons with unique molecular characteristics and developmental profile that are different from corticospinal neurons. They express homeodomain transcription factors including Lhx3 or Chx10 [33, 34]. During development, they are the first supraspinal axons to reach the spinal cord and form discrete groups with distinct anteroposterior and mediolateral locations in the hindbrain [35]. These unique molecular and developmental features may make them more accessible to alterations induced by transgenic expression. In Thy1-YFP transgenic mice, reticuospinal neurons in the brainstem are found to strongly express YFP [36, 37]. This enhanced expression of exogenous protein driven by Thy1 may divert cellular resources so that the expression of intrinsic channel protein such as sodium channels is decreased or its localization is interfered, which may reduce the efficiency of action potential initiation and increase the threshold of TMS-evoked MEPs. Consistently, long-term, high expression of ChR2 protein, through either *in utero* electroporation of CAG::ChR2(H124R)-EYFP-WPRE construct or viral infection driven by αCaMKII promoter, causes morphological abnormalities in axons and somata, suggesting that high ChR2 expression may have a general effect on neuronal morphology [7]. However, the current data do not allow us to exclude other potential changes in neural circuits in this line of transgenic mice; whether neuronal excitability of reticular nuclei is indeed altered requires further investigation.

We did not find significant changes in intrinsic properties of cortical pyramidal neurons, including resting membrane potential, input resistance, and membrane time constant, which is consistent with the finding that the long-latency MEPs, which have a cortical origin, remained unchanged. The normal excitability of cortical pyramidal neurons in ChR2 transgenic mice is also consistent with previous studies that characterized and found unaltered electrophysiological properties in ChR2-expressing neurons. For example, ChR2 expression in cultured hippocampal neurons has no effect on their electrical properties and health [5]. Lentiviral expression of ChR2 for at least 1 week did not cause significant differences in neuronal membrane resistance, resting membrane potential, and voltage change and spike rate in response to current injections [5]. Specific expression of ChR2 at the axon initial segment (AIS) of hippocampal neurons also did not reveal “significant” changes in passive and active membrane properties [6].

Transgenic mice are routinely used in neuroscience research as model systems for visualizing neuronal structures and activity and for manipulating electrophysiological and molecular functions [38–40]. The increased activation threshold for short-latency MEPs in Thy1-ChR2 mice suggests that introducing a new gene into neurons may affect other physiological properties. One striking example is that ChAT–ChR2–EYFP mice carry extra copies of the vesicular acetylcholine transporter gene, which leads to overexpression of the gene VAChT, consequent increased cholinergic tone, and behavioral changes with improved motor endurance and severe cognitive deficits [41]. While obvious phenotypic changes such as the ChAT–ChR2–EYFP mice can be detected, subtler changes would require careful characterization and scrutiny. Therefore, it is important to take into account and control such effects in experimental design and data interpretation. Particularly, comparisons should be made between groups of ChR2-expressing mice with or without any treatment, instead of between groups of WT and ChR2-expressing mice.

In conclusion, we report reduced short-latency MEPs in Thy1-ChR2 transgenic mice, as indicated by the lower MEP amplitude induced by transcranial magnetic and electrical stimulations. Long-latency MEPs and excitability of cortical layer V pyramidal neurons remain intact. The results not only show specific impairment of the motor pathway in this particular line of transgenic mice, but also suggest that transgenic mice may have altered physiological function in addition to the targeted genetic manipulation in general.
